# Effect of Geometrical Structure, Drying, and Synthetic Method on Aminated Chitosan-Coated Magnetic Nanoparticles Utility for HSA Effective Immobilization †

**DOI:** 10.3390/molecules24101925

**Published:** 2019-05-18

**Authors:** Marta Ziegler-Borowska, Kinga Mylkie, Mariana Kozlowska, Pawel Nowak, Dorota Chelminiak-Dudkiewicz, Anna Kozakiewicz, Anna Ilnicka, Anna Kaczmarek-Kedziera

**Affiliations:** 1 Faculty of Chemistry, Nicolaus Copernicus University in Torun, Gagarina 7, 87-100 Torun, Poland; kinga.mylkie@o2.pl (K.M.); nowak19981411@wp.pl (P.N.); dorotachelminiak@wp.pl (D.C.-D.); akoza@umk.pl (A.K.); ailnicka@umk.pl (A.I.); teoadk@umk.pl (A.K.-K.); 2 Karlsruhe Inst Technol, Inst Nanotechnol INT, Hermann von Helmholtz Pl 1, D-76344 Eggenstein-Leopoldshafen, Germany; mari.kozlowska@gmail.com

**Keywords:** aminated chitosan, magnetic nanoparticles, HSA, protein immobilization, molecular dynamics simulations, solvent-free amination

## Abstract

Human serum albumin (HSA) is one of the most frequently immobilized proteins on the surface of carriers, including magnetic nanoparticles. This is because the drug–HSA interaction study is one of the basic pharmacokinetic parameters determined for drugs. In spite of many works describing the immobilization of HSA and the binding of active substances, research describing the influence of the used support on the effectiveness of immobilization is missing. There are also no reports about the effect of the support drying method on the effectiveness of protein immobilization. This paper examines the effect of both the method of functionalizing the polymer coating covering magnetic nanoparticles (MNPs), and the drying methods for the immobilization of HSA. Albumin was immobilized on three types of aminated chitosan-coated nanoparticles with a different content of amino groups long distanced from the surface Fe_3_O_4_-CS-Et(NH_2_)_1–3_. The obtained results showed that both the synthesis method and the method of drying nanoparticles have a large impact on the effectiveness of immobilization. Due to the fact that the results obtained for Fe_3_O_4_-CS-Et(NH_2_)_2_ significantly differ from those obtained for the others, the influence of the geometry of the shell structure on the ability to bind HSA was also explained by molecular dynamics.

## 1. Introduction

Rapid progress in nanotechnology, especially in the design and synthesis of new materials, significantly affects the development of many branches of science and technology [1]. Novel applications of nanomaterials in biomedical and pharmaceutical fields are of particular importance due to the possible high impact on social health issues. Drug delivery processes, diagnostics, catalysis, and pharmaceutical analysis provide good motivation for the production of materials of specific desired characteristics that open up new possibilities and allow for the avoidance of known problems and the disadvantages of traditional old techniques. Magnetic nanoparticles (MNPs) constitute a special group of nanomaterials, whose magnetic core can be coated with both low- and macro-molecular compounds [2]. This coating leads to a material with surface properties designed precisely for its intended use and also easily conducted to the target place, for instance in a living organism, and effortlessly separable from the reaction mixture because of their superparamagnetic properties [3,4]. One of the most common applications of MNPs is a support for the immobilization of catalysts, drugs, and bioligands such as proteins [5,6,7,8]. Nanoparticles designed for this purpose must possess either a surface modified for an effective physical adsorption or enriched in reactive groups prone to forming covalent bonds in the case of chemical immobilization of a ligand [9,10,11].

Because of the difficulty of working with proteins in their free form, MNPs are widely applied for their binding. Good separation of proteins from the reaction mixture requires intensive centrifugation, dialysis, or other more advanced methods [12]. The immobilization of protein on the surface of MNPs allows its separation from the supernatant to be straightforward by applying a magnet. The simplicity and effectiveness of protein separation from the supernatant is of great importance, for example for determination of pharmacokinetic parameters of drugs, such as the degree of binding of the active substance to plasma proteins. Blood plasma contains a number of proteins, of which the most important is human serum albumin (HSA): about 50 wt% [13]. This protein plays a key role in the transport of exo- and endogenous ligands present in the blood, and thus, also in the binding of drugs [14]. 

The drug molecule that is associated with HSA is pharmacologically inactive. As long as it is associated with the protein, it cannot exhibit pharmacological activity and is not metabolizable. For this reason, it is very important to study the in vitro degree of binding of the active substance to HSA. Due to the aforementioned complicated and troublesome work with the free form of the protein, the growing interest can be noticed in investigations of the impact of HSA immobilization on the support surface [15,16]. The amount of HSA bounded to the support should be relatively large in order to achieve the noticeable influence with drugs. The most popular method of binding HSA to the support surface is a covalent immobilization using the carboxylic groups of the protein and the carrier rich in primary amino groups. For this reason, as well as for its biocompatibility and moderate hydrophilicity, chitosan appears to be a great coating for magnetic nanoparticles prepared for HSA covalent binding [17]. 

Despite the knowledge of the above-mentioned advantages, which arise from the immobilization of HSA on a precisely designed support, there is still a lack of detailed reports within the literature that carefully describe and analyze both the methods of synthesis and the structural conditions of support for HSA immobilization performance. In the literature one can find several works that try to explain the geometrical structure of chitosan using theoretical methods [18,19,20]. 

Therefore, the current contribution considers the synthesis of three types of magnetic nanoparticles coated with chitosan enriched with amino groups by a chemical modification. The obtained materials differ with one another in the content of the surface free amino groups which are able to form a covalent bond with HSA. Further on, albumin is immobilized covalently on the obtained nanoparticles and the impact of the method of synthesis and drying of the material on the effectiveness of immobilization is investigated. The extensive experimental analysis of the performance of the MNPs designed for this particular purpose is supported, with the advanced classical molecular dynamics simulations of the models of modified chitosan chains in water. These calculations allow for explanations of the differences in the performance of the three proposed materials. 

## 2. Results and Discussion

### 2.1. Magnetic Nanoparticles Synthesis 

**Figure 1:**
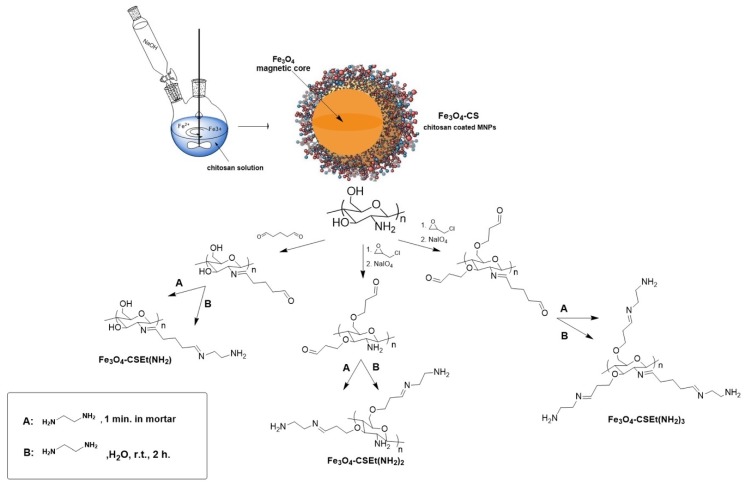
Scheme of the synthesis of aminated chitosan-coated nanoparticles Fe_3_O_4-_CSEt(NH_2_)_1–3._

It is well-known that chitosan coated on a magnetic core keeps free amino groups on the surface [21]. The amount of these groups on the pure unmodified chitosan-coated nanoparticles vary between 2.4 [22] and 3.73 mM/g [23] of material, which may be insufficient for good binding of proteins in the case of biomedical applications. Moreover, the amino groups attached to the pyranose unit in the unmodified chitosan are arranged close to the ring, which may affect the low efficiency of binding of bioligands such as proteins. Enrichment of chitosan into amino groups distanced from the polymer matrix, and in the consequence from the nanoparticles’ surface, will probably make the nanomaterial able to bind more protein than unmodified chitosan. The synthetic strategy for the preparation of magnetic nanoparticles Fe_3_O_4_-CS-Et(NH_2_)_1–3_ coated with aminated chitosan preparation is presented in Figure 1. 

The first step for both of the procedures used was a standard in situ functionalization of Fe_3_O_4_ magnetic core with chitosan molecules via the co-precipitation method. Next, the different reactivity of amino and hydroxyl groups of chitosan was exploited. The key step in this synthesis was the amination reaction of glutaraldehyde-activated chitosan. The reaction was carried out using two methods: traditionally in a solvent and a newly developed solvent-free method, performed by pounding in mortar for 1 minute [24]. After amination, three types of nanoparticles were prepared Fe_3_O_4-_CSEt(NH_2_)_1–3._ Before nanoparticles synthesis, as a preliminary study pure chitosan was modified and the most effective method for chitosan core modification was chosen [25]. 

The obtained magnetic nanoparticles were dried using two methods: in a vacuum oven at 35 °C and by lyophilization, to investigate whether the drying method could affect the material parameters and its utility for HSA binding.

### 2.2. Characterization of Prepared Magnetic Nanoparticles and Theoretical Calculation

**Figure 2:**
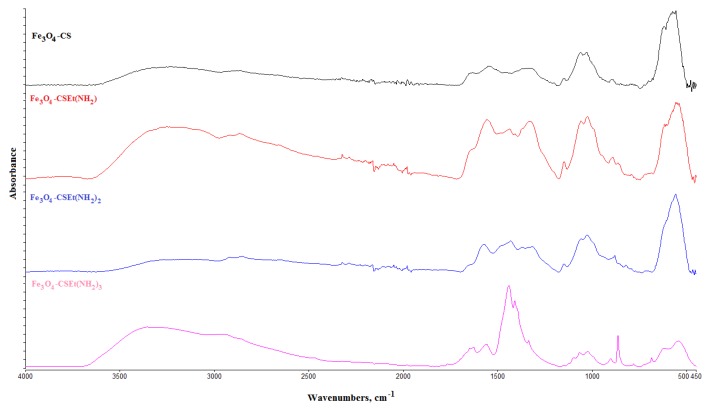
ATR-FT IR results for the prepared nanoparticles.

The structure of the obtained materials was confirmed by ATR-FT IR spectroscopy. The spectra obtained for the nanoparticles prepared via two methods and dried in two ways were identical. Figure 2 shows the representative spectra for magnetic nanoparticles. 

The C = N and N-H characteristic vibration peaks at 1630 and 1540 cm^−1^ were observed and increased with the C = N and primary amine groups’ formation in prepared nanoparticles. The peaks at 1400 cm^−1^ and at 860 cm^−1^ and 766 cm^−1^ increased with the growing number of N-H stretching and N-H wagging vibrations in primary amine groups. These peaks were not observed in nanoparticles coated with pure chitosan (Fe_3_O_4_-CS). The signal at 571 cm^−1^ was assigned to the Fe-O group of magnetite. 

Each of the prepared nanoparticles contains primary amino groups moved away from the chitosan chain, which should make them much more available for bioligands such as enzymes and could serve as a qualitative measure of bioactivity. The amount of free amino groups on the surface of the obtained nanoparticles is within the range from 3.15 to 8.34 mM/g (Table 1). As predicted, most amine groups are on the surface of nanoparticles coated with modified chitosan containing the three amino groups distanced from the polymer chain. In addition, it should be noted that both the synthesis and drying methods did not affect the number of free groups on the surface of the nanoparticles.

**Figure 3:**
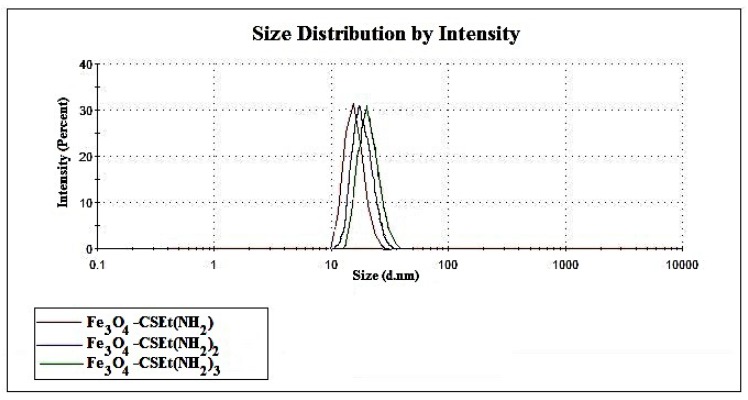
The distribution of the prepared nanoparticles’ size.

The size of the obtained nanoparticles was in the range from 22 to 29 nm (Table 1) and similarly, as with the number of free amino groups, it did not depend on the method of synthesis or drying. Moreover, as was expected, the high number of amino groups distanced from the surface results in higher nanoparticles’ size, but the differences are not spectacular. The dynamic light scattering (DLS) analysis also demonstrated that the prepared nanoparticles were rather homogenous in sizes (Figure 3). 

The primary adsorption data were the basis for calculation of the so-called structural parameters, i.e., specific surface area and pore-size distribution. The nitrogen isotherms resulted in type-IV shape [26]. The hysteresis loop is type H2 (according to IUPAC classification) in the range of 0.3–0.98 relative pressure. H2 hysteresis contain more complex pore networks consisting of pores with ill-defined shape and wide pore-size distribution. These results suggest that the Fe_3_O_4_-CS-Et(NH_2_)_1–3_ nanomaterials are characterized by mesoporous structure. It was expected that the surface area of the prepared nanoparticles would depend on the amount of amino groups.

**Figure 4:**
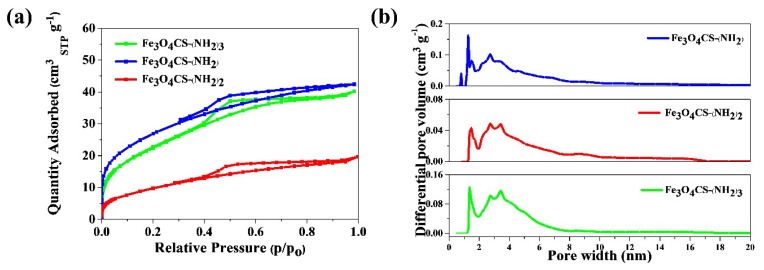
(**a**) Nitrogen adsorption–desorption isotherms (**b**) DFT pore volume distributions of the Fe_3_O_4-_CSEt(NH_2_)_1–3_ nanoparticles.

The maximum surface area, as it is shown in Table 1, was determined as 98 m^2^ g^−1^ and 84 m^2^ g^−1^ for the Fe_3_O_4_-CS-Et(NH_2_) and Fe_3_O_4_CS-Et(NH_2_)_3_ samples, respectively. For these two types of nanoparticles, increasing the contents of free amino groups causes a considerable decrease in the specific surface area, as calculated by the Brunauer–Emmett–Teller (BET) surface adsorption method. The material Fe_3_O_4_-CS-Et(NH_2_)_2_ had the lowest surface area—34 m^2^ g^−1^, which was unexpected at this stage of the study. The pore-size distribution curves were calculated by using the density functional theory (DFT) method, and are depicted in Figure 4b. The pore size distribution (PSD)results can be interpreted in two ways. (1) The run of PSD functions may be attributed to the presence of micro- and mesopores. Mesopores contribute meaningfully to the total porosity, and the total porosity share of mesopores to the total volume increases, as is shown in Table 1. (2) The relatively low surface area and the observed (HR-TEM) globular structure of the materials suggest that inter-granule spaces may contribute to the total pore volume as well. Such a phenomenon was observed for Aerosil, which is non-porous silica, however it exhibits the hysteresis loop on adsorption–desorption isotherms [27].

The effect of the drying method on the adsorption–desorption isotherms measurement results for synthesized materials can be neglected, due to the measurement technique including a high vacuum operation and a wide temperature range. No differences were noted in the results obtained for materials synthesized by amination with and without solvent.

**Figure 5:**
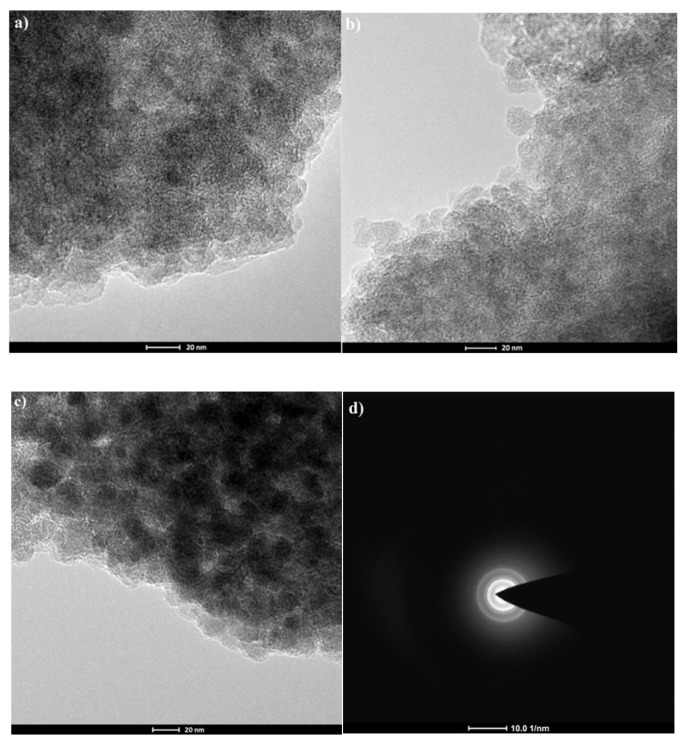
Transmission electron microscope (TEM) pictures of prepared magnetic nanoparticles (MNPs), (**a**) Fe_3_O_4_CS-Et(NH_2_), (**b**) Fe_3_O_4_CS-Et(NH_2_)_2_, (**c**) Fe_3_O_4_CS-Et(NH_2_)_3_, and (**d**) selected area diffraction pattern (SADP) for MNPs.

The results of transmission electron microscope (TEM) analysis are demonstrated in Figure 5. The pictures confirm that the magnetite core has been coated with a polymer layer, the obtained particles had a size in the range 22–29 nm (Figure 5a–c), and the particles are aggregated. To determine the structure of the magnetic core the electron diffraction was applied. The typical selected area diffraction pattern (SADP) from the group of particles is shown in Figure 5d.

The crystal structure and phase purity of the prepared nanoparticles were also verified by X-ray Diffraction (XRD) analysis. Six characteristic peaks for Fe_3_O_4_, marked by their indices ((2 2 0), (3 1 1), (4 0 0), (4 2 2), (5 1 1), (4 4 0)), were observed for all samples. These peaks are consistent with the X’Pert High Score database and reveal that the resultant nanoparticles core was pure Fe_3_O_4_ with a spinael structure. This also confirmed that the co-precipitation coating of magnetite nanoparticles did not cause the phase change of Fe_3_O_4_.

**Figure 6:**
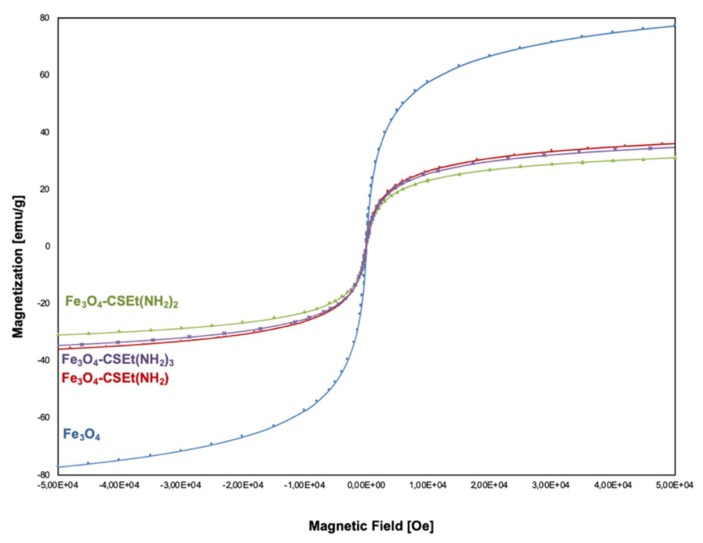
Magnetization hysteresis curve for aminated chitosan-coated nanoparticles Fe_3_O_4_-CSEt(NH_2_)_1–3_ and naked nanoparticles (Fe_3_O_4_).

The magnetization measurements of aminated chitosan-coated nanoparticles were examined using the quantum design Superconducting Quantum Interference Device (SQUID) technique in the employed magnetic field, between −8000 to +8000 Oe, at room temperature. The results of magnetization measurements (T = 300 K) as M versus H curve, are presented in Figure 6. The value of saturation of magnetization of naked Fe_3_O_4_ nanoparticles is about −77 emu/g. For nanoparticles coated with modified chitosan, regardless of the method of synthesis and drying, a decrease in the value of saturation of magnetization to –35, –34, and –30 emu/g is observed respectively, for Fe_3_O_4_-CSEt(NH_2_), Fe_3_O_4_-CSEt(NH_2_)_2_, and Fe_3_O_4_-CSEt(NH_2_)_3_ nanoparticles. This result is comparable to the data available in the literature for naked and monolayer-coated magnetic nanoparticles. In addition, it can be noted that no significant differences in magnetization can be seen between the prepared materials. Nanoparticles coated with chitosan with two matrix-separated amino groups (Fe_3_O_4_-CS-Et(NH_2_)_2_) showed slightly lower magnetization, however the differences are not spectacular.

In order to better understand the nature of the surface of the polymer shell covering the magnetic core of the obtained nanoparticles, a contact angle measurement was made for films obtained from modified chitosan without the addition of magnetite. The obtained results are shown in Table 2. For comparison, the contact angle measurement for pure unmodified chitosan (CS) was also performed [28]. As can be seen, the surface of chitosan containing one (CS-Et(NH_2_)) and three (CS-Et(NH_2_)_3_) amino groups removed from the surface is very similar to pure chitosan in terms of hydrophilicity. Moreover, it appears that the surface of these polymers is slightly more hydrophobic, as indicated by the polar component (γ_p_) values for pure chitosan 3.23 and 2.66 for CS-Et(NH_2_), and 2.32 for CS-Et(NH_2_)_3_, respectively. The results obtained for CS-Et(NH_2_)_2_ are unexpected. The contact angle for this material appears to be significantly smaller than for the others, which suggests the higher hydrophilicity. Additionally, the surface of this material is characterized by the highest value of the polar component (γ_p_ = 8.10), which indicates the presence of a greater number of hydrophilic groups on the surface of the material than in the case of the previous modified chitosan.

The observed differences are directly related to the molecular structuring of the modified polymers arising from their different chemical compositions. Apparently, in the case of CS-Et(NH_2_)_2_ polymer, the effect of preferable configuration of two distanced amino groups on the surface may favor the hydrogen bonds formation between these groups and hydrophilic test liquid (glycerin) [29]. It results in better wettability of material to polar solvents. A similar tendency was also observed in our previous studies [25].

**Figure 7:**
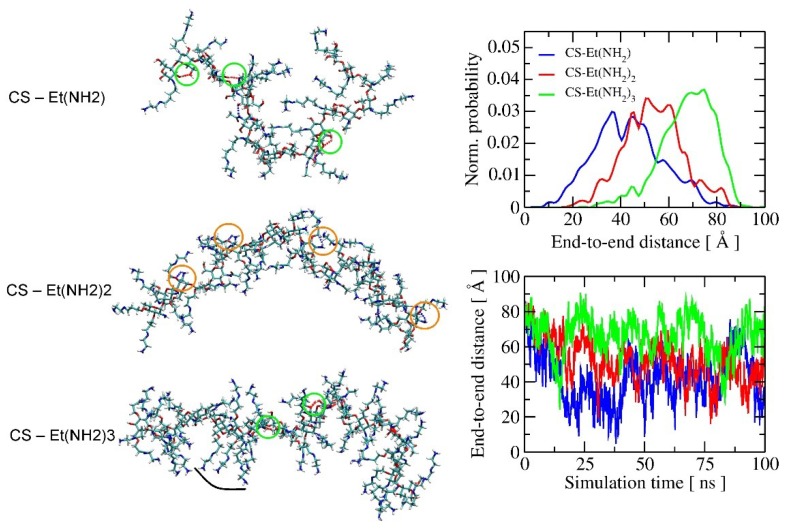
Structuring of modified chitosan polymers with molecular dynamics simulations using the extended CHARMM36 force field for carbohydrates. The selected snapshots of CS-Et(NH_2_)_1–3_ conformations are shown in the panel on the left, while the flexibility/stiffness of the polymer chains as a function of the end-to-end distance is shown in the panel on the right. A description of the computational setup is provided in the Section 3.2.5.: computational details.

In order to get an insight into the molecular structuring of the investigated polymers, molecular dynamics (MD) simulations were carried out using an extended CHARMM36 carbohydrate force-field of the fragment (twenty repeated units) of the chitosan-modified polymers in water at room temperature (see Section 3.2.5.: computational details). During 100 ns of MD simulations, a significant difference in polymer dynamics in water was observed, influenced mainly by intramolecular hydrogen bonds interactions of a diverse nature. In most cases, H-bonds occur close to the polymer backbone, inducing structural twists and, therefore, different polymer flexibility. CS-Et(NH_2_) polymer has only one modified side chain and is rich in hydroxyl groups. As a result, its structuring is caused by strong O-H···O H-bonds (marked with green circles in Figure 7). 

This polymer has the highest flexibility among other polymers, as was confirmed by the analysis of its end-to-end distance, as shown in the panel on the right in Figure 7—the possible chain length can vary from less than 10 to about 90 Å. At the same time, CS-Et(NH_2_) tends to form diverse temporary aggregates of a random architecture. This causes high probability of local pores formation and indicates a possible high surface area, as was seen in adsorption–desorption isotherms measurements. O-H···O H-bonds are also often formed during the structuring of CS-Et(NH_2_)_3_ (see bonds marked with green circles in Figure 7). However, here the main source of the formed H-bonds are chitin units, which were placed in the model of the chitosan polymer at every forth monomer, consistent with approximately 80% of the degree of deacetylation of the polymers used in this experiment. Due to the fact that CS-Et(NH_2_)_3_ has all three side chains modified, polymer is the most rigid (see end-to-end distance in Figure 7), but described H-bonds induce the additional degree of freedom and cause the formation of domains of long side chains after every chitin unit (shown with the black curve in Figure 7). Moreover, the tendency of large pore formation was also found in the case of CS-Et(NH_2_)_3_, but due to the relatively small size of the polymer model, one cannot observe the whole pore formed. Nonetheless, it may also explain the high surface area of this polymer and the increase in its hydrophobicity (see Table 2).

Structuring of the CS-Et(NH_2_)_2_ polymer is induced by a different type of H-bonding, i.e., N-H···O, which is marked in orange circles in Figure 7 for clarity. N-H···O H-bonds, formed near the polymer backbone, were not observed for CS-Et(NH_2_) and CS-Et(NH_2_)_3_, as the respective amino group was modified by a longer amino side chain. The dense existence of the unmodified amino group causes the formation of lots of strong N-H···O H-bonds during MD simulation. It makes the polymer structuring more synchronized and causes the formation of denser polymer aggregates. This has a double effect: firstly, it lowers the reactive surface area of the polymer to 34 m^2^/g, and secondly, it induces higher localization of amino groups on the surface, which causes an increase of the polarity of the surface (Table 2). The observed phenomena are of a complex nature and require a more detailed analysis, which will be within the scope of our further investigations.

### 2.3. HSA Covalent Immobilization on the Prepared Nanoparticles Surface

**Figure 8:**
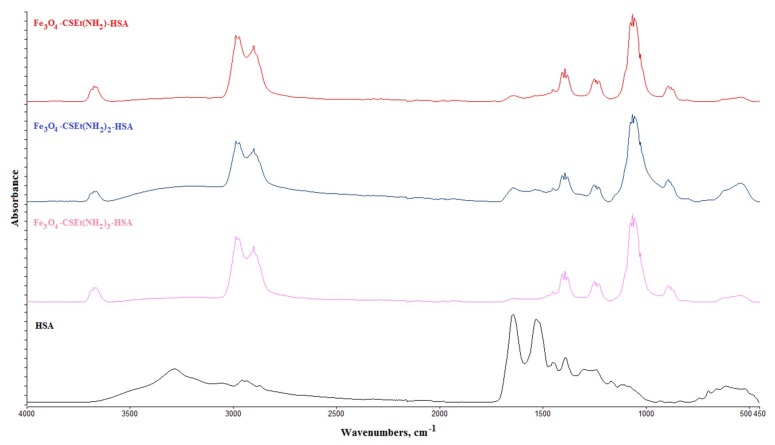
ATR-FT IR results for human serum albumin (HSA)-coated nanoparticles.

Immobilization of HSA was carried out under identical conditions for all obtained magnetic nanoparticles, with the division of materials into a method of synthesis and drying. EDC/NHS was chosen as a protein binding agent and the incubation was carried out at 21 °C in phosphate buffer PBS (pH = 6.5). The covalent binding of HSA to the surface of the nanoparticles was confirmed by ATR-FT IR analysis (Figure 8). The ATR-FT IR spectra after HSA immobilization (Figure 8) showed strong modifications in comparison with spectra obtained prior to the immobilization process (Figure 2). The most visible changes appeared in the 3600 cm^−1^, 3000–2800 cm^−1^, and about 1150 cm^−1^ range. The disappearance of the broad band derived from amine groups (3300–3500 cm^−1^) in the nanoparticles without has, and the formation of a narrow band at 3600 cm^−1^, confirms the binding of HSA by amide bonds. The new band observed at 1150 cm^−1^, for nanoparticles with bonded HSA, arises from the stretching vibration of C-O in the structure of protein. Bands of about 1250 and 1400 cm^−1^ were assigned to a combination of N-H and C-N vibrations. Clear bands in the range of 3000–2800 cm^−1^ additionally confirm the covalent binding of protein to the surface of the nanoparticles [30].

The amount of immobilized HSA bounded on magnetic nanoparticles’ surface was determined by the Bradford protein assay method after magnetic nanoparticles separation by applying a magnet [31]. The results of HSA immobilization obtained for all nanoparticles are presented in Table 3 and they are very good compared to those found in the literature [32].

**Figure 9:**
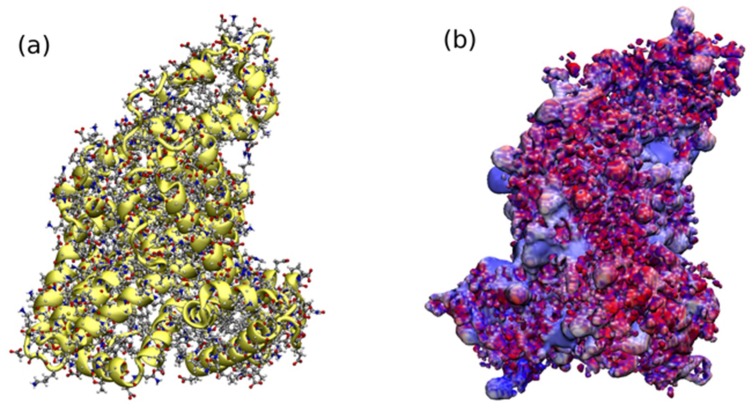
Visualization of the human albumin protein (pdb number 1AO6) in the protonation state at pH = 7. The structure of the protein in all atom (Licorice) and helical (NewCartoon) representation with shown outer functional groups (**a**). Electrostatic potential map of the protein [33] (**b**) depicts places in red and blue corresponding to the negatively and positively charged regions, respectively. Electrostatic potential was calculated using Adaptive Poisson-Boltzmann Solver (APBS) [34] with a default grid dimension, as implemented in APBS software. Hydrogen atoms and charges were placed according to pKa of amino acids using the CHARMM protein force field, and assigned protonation states at pH = 7 with PROPKA software, as implemented in the PDB2PQR server [35]. The visualization was made in VMD software using the RWB color scheme for the electrostatic potential map with isovalue 2 for the charge range (–3–+3).

As can be seen, the method of amination of chitosan coated on nanoparticles’ surface, as well as the method of drying after synthesis, had a large impact on the efficiency of HSA immobilization in the case of two types of material: Fe_3_O_4_-CSEt(NH_2_) and Fe_3_O_4-_CSEt(NH_2_)_3_ nanoparticles. The solvent-free amination reaction resulted in an increase in the efficiency of albumin immobilization for nanoparticles, both dried in the vacuum drier and by lyophilization. In the case of nanoparticles dried in a vacuum dryer for materials obtained without a solvent, an increase by about 35% was observed in the immobilization yield. However, the drying method had the greatest influence on the amount of immobilized albumin for these two nanomaterials, (Fe_3_O_4_-CSEt(NH_2_) and Fe_3_O_4-_CSEt(NH_2_)_3_). In comparison with the results obtained for nanoparticles dried in a vacuum dryer, regardless of the method of synthesis, the amount of albumin immobilized on nanoparticles dried by lyophilization was about 200% higher. Therefore, it can be concluded that while the solvent-free method of synthesis increases the yield of immobilization slightly, the method of drying nanoparticles has a key influence on the protein binding efficiency. This is most likely due to the more effective removal of the solvent which interacted with the amino groups of the aminated chitosan shell on the principle of hydrogen bonds. The geometrical structure of the polymer shell coated on nanoparticles also explains the immobilization results obtained for Fe_3_O_4-_CSEt(NH_2_)_2_ nanoparticles. For this material, the amount of bounded HSA was the smallest. Furthermore, both the synthesis and drying methods do not affect the HSA immobilization efficiency in this case. According to the molecular dynamics simulations, CS-Et(NH_2_)_2_ has the densest type of aggregate due to the presence of a free unsubstituted amino group close to the backbone, and therefore, has the lowest surface area available for protein binding. A higher density of amino groups on the surface of the coated polymer shell has a minor influence on the protein binding affinity due to the large molecular size of HSA (shown in Figure 9) and the more difficult protein fitting to the functional groups located on the dense polymer aggregate of CS-Et(NH_2_)_2_. 

Binding of HSA takes place using its carboxyl and amino groups on the surface of nanoparticles. As can be seen in Figure 9, carboxyl groups are highly populated on the surface of HSA, which in general causes the total negative charge of the protein at a neutral pH. The efficiency of immobilization is therefore dependent to a large extent on the availability of free amino groups on the surface of the nanoparticles, and the available surface area of the coated polymer. Since the total surface area of Fe_3_O_4_-CSEt(NH_2_)_2_ nanoparticles is lower, the amino groups on the surface cannot be actively involved in the forming of amide bonds with the carboxyl groups on the surface of HSA, therefore, its binding is hindered and less protein immobilizes.

In the case of Fe_3_O_4_-CSEt(NH_2_) and Fe_3_O_4_-CSEt(NH_2_)_3_ nanoparticles, an increase in the amount of bound HSA can be observed as the polymers possess more available surface areas and the number of interacting amino groups on the surface of the nanoparticles increases. However, differences in the amount of bound HSA for NH_2_ groups (3.15 mM/g) in the Fe_3_O_4_-CSEt(NH_2_) and Fe_3_O_4_-CSEt(NH_2_)_3_ (8.34 mM/g) are not as large as one would expect. Presumably, in Fe_3_O_4_-CSEt(NH_2_)_3_ nanoparticles due to the large size of the HSA molecule, the amount of amide bonds formed during the immobilization is smaller than the amount of NH_2_ groups on the nanoparticles’ surface.

It is known that the covalent immobilization of HSA can lead to structural changes in the protein, and can even involve partial degradation. These changes may result in a lack of HSA activity and, consequently, the low utility of such a material, for example for the determination of pharmacokinetic parameters of drugs. Immobilization by carboxyl groups on the surface of HSA should not lead to protein deactivation due to the fact that the drug binding sites through this protein are located in the depth of its structure, i.e., in the places with positively charged pockets (Figure 9). In order to investigate whether immobilized HSA retained its activity, the anti-HSA aggregation test was performed for HSA-coated nanoparticles (Fe_3_O_4_-CSEt(NH_2_)-HSA, Fe_3_O_4_-CSEt(NH_2_)_2_-HSA, and Fe_3_O_4_-CSEt(NH_2_)_3_-HSA) on the test glass slide for 10 μL of nanoparticles and 10 μL of anti-HSA in PBS (pH = 7). A control test for nanoparticles without HSA was also accomplished. In the test for HSA-coated nanoparticles, a clear aggregation was observed, which indicates the preserved activity of albumin after immobilization. No aggregation was noticed for the control. The fact that the immobilized HSA retained its activity allows for the possibility of using these materials in pharmaceutical analysis or in determining the pharmacokinetic parameters of the drugs.

In summary, both the synthesis and drying methods have an effect on the HSA immobilization yield only for Fe_3_O_4_-CSEt(NH_2_) and Fe_3_O_4_-CSEt(NH_2_)_3_ materials. Increased immobilization efficiency is observed in the case of non-solvent amination. The drying method has a key influence on the effectiveness of HSA binding by nanoparticles: the immobilization of the protein on the surface of the dried materials by lyophilization is 200% higher than on the same nanoparticles dried in a vacuum oven. The Fe_3_O_4_-CSEt(NH_2_)_2_ nanoparticles deviate from the rest of the materials, which is explained by the geometrical structure of the aminated chitosan-coated on magnetite core, whose unsubstituted amino groups close to the backbone create intramolecular hydrogen bonds, aggregating more closely and hindering the reaction with HSA.

## 3. Materials and Methods 

### 3.1. Materials and Reagents 

Iron (II) chloride tetrahydrate, iron (III) chloride hexahydrate, chitosan (low molecular weight), glutaraldehyde, sodium periodate, ethylenediamine, acetic acid, sodium hydroxide, EDC (*N*-(3-dimethylaminopropyl)-*N*′-ethylcarbodiimide hydrochloride), sulfo-NHS (*N*-hydroxy- sulfosuccinimide sodium salt), glycine, isopropanol, acetic acid, ethanol, ninhydrin reagent and Bradford reagent, Human Serum Albumin (HSA), and anti-HSA were purchased from Sigma–Aldrich (Darmstadt, Germany) and used without further purification. Solvents, sodium phosphate dibasic dehydrate, and orthophosphoric acid solution for phosphate buffer (PBS) preparation, sodium acetate were purchased from POCh Gliwice (Gliwice, Poland). All solutions were prepared with deionized water. 

### 3.2. Methods 

#### 3.2.1. Analysis and Characterization

##### Attenuated Total Reflectance Fourier Transform Infrared (ATR-FTIR) 

The structure of the prepared nanoparticles was characterized with the Attenuated Total Reflectance Fourier Transform Infrared (ATR-FTIR)–Spectrum Two^TM^ (Perkin Elmer, Waltham, MA, USA). Spectra were recorded over the region from 4000 to 450 cm^−1^, at a resolution of 4 cm^−1^, 32 scans were performed at room temperature. 

##### Scanning and Transmission Electron Microscopy (SEM, TEM)

A scanning electron microscope (SEM) 1430 VP LEO Electron Microscopy Ltd. was applied. The morphology and size of the prepared nanoparticles were characterized by transmission electron microscope (TEM) Tecnai F20 X-Twin, FEI Europe, equipped with energy dispersive X-ray spectrometer (EDX) Edax. Nanoparticles were dispersed in ethanol with the concentration of 1mg/mL and treated with an Inter Sonic IS-1K bath (95 W for 15 min) and dropped onto holey carbon-coated copper grids covered with a perforated carbon film. Observation was performed at 200 kV. 

##### Dynamic Light Scattering (DLS)—Particles Size Analysis

Dynamic light scattering (DLS) measurements were performed using a Malvern Nano Zetasizer ZS90 instrument (Malvern, UK). Measurements were performed at a wavelength of 633 nm, using a detection angle of 25 °C. All samples were purified by filtration (0.45 mm PTFE filter). All measurements were triplated and the reported values are the mean diameter. 

##### X-ray Diffraction (XRD) Measurement 

X’PERT Pro Philips Diffractometer (Cu Kα1, wavelength 1.54056 Å, range 2Theta 5–90°, scan step size 0.020 °, time per step 3.00 s) was used for X-ray diffraction (XRD) measurement.

##### Magnetization Measurement

Magnetic measurements (in solid state) were carried out using a Quantum Design Superconducting Quantum Interference Device (SQUID) magnetometer (model MPMS3, San Diego, CA, USA) in Laboratory of Magnetic Measurements, Faculty of Chemistry, University of Wroclaw (Poland). M/H curves were recorded in the temperature (T) 298 K.

##### Contact Angle Measurement

The hydrophilic/hydrophobic properties of pure polymers were analyzed by contact angle measurements using a DSA G10 goniometer (Kruss GmbH, Hamburg, Germany). A drop of glycerin or diiodomethane was placed on the polymer film surface with a micro syringe. The sessile drop image was recorded and digitized by camera. The drop shape analysis and determination of the contact angle were done with help of instrument software. The reported contact angle (wettability angle) value was on average at least 10 measurements for each specimen. All measurements were carried out at room temperature.

#### 3.2.2. Magnetic Nanoparticles Synthesis

##### Magnetic Nanoparticles Coated with Modified Chitosan with One Long-Distanced Free Amino Group (Fe_3_O_4_ CS-Et(NH_2_))—Traditional Chitosan Amination in Solution

Chitosan (0.2 g) was added to acetic acid solution (C = 1%, 20 mL) and mechanically stirred at room temperature for 20 min. Iron (II) chloride tetrahydrate (0.74 g, 3.75 mmol), and iron (III) chloride hexahydrate (2.02 g, 7.5 mmol) were added (1:2 molar ratio) and the resulting solution was chemically precipitated at room temperature by adding dropwise 30% solution of NaOH (7mL). The black mixture was formed, separated by applying a magnet, and washed with deionized water five times. Then, 10 mL of bicarbonate buffer pH = 10 and 10 mL of glutaraldehyde solution (5%) were added and the mixture was mechanically stirred at room temperature for 1 h. Next, 20 mL of aqueous solution of ethylenediamine (2.4 g, 40 mmol) was added and the mixture was stirred at room temperature for the next 2 h. The resulting magnetic material was recovered from the suspension by applying a magnet, washed five times with deionized water, and dried under a vacuum at 35 °C for 24 h (V) or by lyophilization (L) 

##### Magnetic Nanoparticles Coated with Modified Chitosan with Two Long-Distanced Free Amino Groups (Fe_3_O_4_ CS-Et(NH_2_)_2_)—Traditional Chitosan Amination in Solution

Chitosan (0.2 g) was added to acetic acid solution (C = 1%, 20 mL) and mechanically stirred at room temperature for 20 min. Iron (II) chloride tetrahydrate (0.74 g, 3.75 mmol), and iron (III) chloride hexahydrate (2.02 g, 7.5 mmol) were added (1:2 molar ratio) and the resulting solution was chemically precipitated at room temperature by adding dropwise 30% solution of NaOH (7mL). The black mixture was formed, epichlorohydrin (0.2 mL, 2.5 mmol) was added, and the mixture was stirred at 50° for 2 h. After cooling to room temperature, the sodium periodate solution (0.16 g in 2.5 mL of water) was added and the mixture was stirred for 30 min. The 20 mL of aqueous solution of ethylenediamine (2.4 g, 40 mmol) was added and the mixture was stirred at room temperature for 2h. The resulting magnetic material was recovered from the suspension by applying a magnet, washed five times with deionized water, and dried under a vacuum at 35 °C for 24 h (V) or by lyophilization (L). 

##### Magnetic Nanoparticles Coated with Modified Chitosan with Three Long-Distanced Free Amino Groups (Fe_3_O_4_ CS-Et(NH_2_)_3_)—Traditional Chitosan Amination in Solution

Chitosan 0.2 g was added into 1% acetic acid solution (20 mL) and mechanically stirred at room temperature for 20 min. Iron (II) chloride tetrahydrate (0.74 g, 3.75 mmol), and iron (III) chloride hexahydrate (2.02 g, 7.5 mmol) were added (1:2 molar ratio) and the resulting solution was chemically precipitated at room temperature by adding dropwise 30% solution of NaOH (7mL). The black mixture was formed, epichlorohydrin (0.2 mL, 2.5 mmol) was added, and the mixture was stirred at 50° for 2 h. After cooling to room temperature, the sodium periodate solution (0.16 g in 2.5 mL of water) was added and the mixture was stirred for 30 min. The black precipitate was separated by filtration and washed by deionized water five times. Then 10 mL of bicarbonate buffer pH =10 and 10 mL 5% glutaraldehyde solution were added and the mixture was mechanically stirred at room temperature for 1 h. Next, 20 mL of aqueous solution of ethylenediamine (2.4 g, 40 mmol) was added and the mixture was stirred at room temperature for 2h. The resulting magnetic material was recovered from the suspension by applying a magnet, washed five times with deionized water, and dried under a vacuum at 50 °C for 24 h (**V**) or by lyophilization (**L**). 

##### Magnetic Nanoparticles Coated with Modified Chitosan with One Long-Distanced Free Amino Group (Fe_3_O_4_-CSEt(NH_2_))—Solvent Free Chitosan Amination 

Chitosan (0.2 g) was added to acetic acid solution (C = 1%, 20 mL) and mechanically stirred at room temperature for 20 min. Iron (II) chloride tetrahydrate (0.74 g, 3.75 mmol), and iron (III) chloride hexahydrate (2.02 g, 7.5 mmol) were added (1:2 molar ratio) and the resulting solution was chemically precipitated at room temperature by adding dropwise 30% solution of NaOH (7mL). The black mixture was formed, separated by applying a magnet, and washed by deionized water five times. Then, 10 mL of bicarbonate buffer pH = 10 and 10 mL of glutaraldehyde solution (5%) were added and the mixture was mechanically stirred at room temperature for 1 h, separated by a magnet, and dried under a vacuum. Magnetic nanoparticles were pounded with ethylenediamine (2.4 g, 40 mmol) in an agate mortar at room temperature for 1 min without solvent. The resulting magnetic material was washed five times with deionized water and dried under a vacuum at 35 °C for 24 h (**V**) or by lyophilization (**L**). 

##### Magnetic Nanoparticles Coated with Modified Chitosan with Two Long-Distanced Free Amino Group (Fe_3_O_4_-CSEt(NH_2_)_2_)—Solvent Free Chitosan Amination

Chitosan (0.2 g) was added to acetic acid solution (C = 1%, 20 mL) and mechanically stirred at room temperature for 20 min. Iron (II) chloride tetrahydrate (0.74 g, 3.75 mmol), and iron (III) chloride hexahydrate (2.02 g, 7.5 mmol) were added (1:2 molar ratio) and the resulting solution was chemically precipitated at room temperature by adding dropwise 30% solution of NaOH (7mL). The black mixture was formed, epichlorohydrin (0.2 mL, 2.5 mmol) was added, and the mixture was stirred at 50° for 2 h. After cooling to room temperature, the sodium periodate solution (0.16 g in 2.5 mL of water) was added and the mixture was stirred for 30 min, separated by a magnet, and dried under a vacuum. Magnetic nanoparticles were pounded with ethylenediamine (2.4 g, 40 mmol) in an agate mortar at room temperature for 1 min without solvent. The resulting magnetic material was washed five times with deionized water and dried under a vacuum at 35 °C for 24 h (**V**) or by lyophilization (**L**). 

##### Magnetic Nanoparticles Coated with Modified Chitosan with Three Long-Distanced Free Amino Group (Fe_3_O_4_-CSEt(NH_2_)_3_)—Solvent Free Chitosan Amination

Chitosan 0.2 g was added into 1% acetic acid solution (20 mL) and mechanically stirred at room temperature for 20 min. Iron (II) chloride tetrahydrate (0.74 g, 3.75 mmol), and iron (III) chloride hexahydrate (2.02 g, 7.5 mmol) were added (1:2 molar ratio) and the resulting solution was chemically precipitated at room temperature by adding dropwise 30% solution of NaOH (7mL). The black mixture was formed, epichlorohydrin (0.2 mL, 2.5 mmol) was added, and the mixture was stirred at 50° for 2 h. After cooling to the room temperature, the sodium periodate solution (0.16 g in 2.5 mL of water) was added and the mixture was stirred for 30 min. The black precipitate was separated by filtration and washed by deionized water five times. Then, 10 mL of bicarbonate buffer pH = 10 and 10 mL 5% glutaraldehyde solution was added and the mixture was mechanically stirred at room temperature for 1 h, separated by a magnet, and dried under vacuum. Magnetic nanoparticles were pounded with ethylenediamine (2.4 g, 40 mmol) in an agate mortar at room temperature for 1 min without solvent. The resulting magnetic material was washed five times with deionized water and dried under a vacuum at 35 °C for 24 h (V) or by lyophilization (L). 

#### 3.2.3. Quantification of Available Primary Amino Groups on Magnetic Nanoparticles Surface

The amount of primary amino groups on the magnetic nanoparticles were estimated by the ninhydrin method. The calibration curve was prepared using glycine as a standard ranging from 0.6 mM to 2 mM in 0.1mM acetate buffer of pH 5.5. The ninhydrin reagent (2 mL) was added to 2 mL solution of each concentration of glycine and was well mixed. The blank reagent was composed with 2 mL of distilled water and 2 mL of ninhydrin reagent. The suspensions of magnetic nanoparticles were obtained by dispersing of 10 mg of synthesized nanoparticles in 2 mL of 0.1 mM buffer acetate (pH 5.5), and then 2 mL of ninhydrin reagent was added. All standard curve solutions and suspensions of nanoparticles were capped, mixed by hand and heated in boiling water for 15 min. After cooling, 3 mL of 50% ethanol was added to each tube. The concentration of primary amino groups was determined with the use of spectrophotometric measurements at 570 nm.

#### 3.2.4. Human Serum Albumin Immobilization

The EDC (2 mg) was added to 10 mg of HSA in 1mL of 50 mM PBS (pH 6.5) and incubated at 21 °C for 1 h. Then sulfo-NHS (2.4 mg) in 50 μL of 50 mM PBS was added and incubated, as previously (21 °C, 1 h). After incubation magnetic nanoparticles, 50 mg, were added and incubated for 2h at 21 °C. Then the supernatant was removed by applying a magnet and the amount of immobilized HSA bounded on magnetic nanoparticles’ surface was determined by the Bradford protein assay method. A calibration curve (R^2^ = 0.998) constructed with analyzed protein solution of known concentration (0.1–1.1 mg/mL) was used in the calculation of albumin concentration. All data used for calculation are the average of a triplicate of experiments. The activity agglutination test was performed for HSA-coated magnetic nanoparticles on the test glass slide for 10 μL of nanoparticles and 10 μL of HSA solution in PBS (pH = 7). The aggregation ability was estimated. A control test for nanoparticles without HSA was also performed.

#### 3.2.5. Computational Details

Molecular dynamics simulations of three types of modified chitosan were performed using the CHARMM36 force field for carbohydrates [36], extended for parameters of the side chain polymer modifications, and generated using CHARMM General Force Field (CGenFF) program (v.1.0.0) [37,38]. Simulations were performed for the model of polymers consisting of 20 repeated units, where every forth unit was a nonmodified chitin monomer, mimicking the fragment of the polymer synthesized with 80% of deacetylation degree. Polymers were solvated in TIP3P water [39] with minimum layer of water of 30 Å, resulting in total amount of atoms in a system in the range 140,000–150,000 atoms. All simulations were performed at 300 K using the NAMD program package (v2.12b1, GPUs/CUDA) [5,40] using periodic boundary conditions. Each system was firstly minimized in 20,000 steps, heated for 60 ps, and equilibrated for 400 ps. All MD simulations were performed for 100 ns at 1 atm in the NPT ensemble, which was controlled by the Nosé–Hoover Langevin piston method using a damping time constant of 50 ps and a period of 200 ps. The full electrostatic interactions beyond 12 Å were evaluated by the particle mesh Ewald algorithm with a PME grid spacing less than 1 Å. Nonbonding forces were smoothly switched off starting from the distance of 10 Å till the cutoff of 12 Å. The nonbonded neighboring forces were evaluated by the Verlet neighbor list, with a pair-list distance of 13.5 Å. The timestep of MD simulations was set to 2 fs. All equations of motion were integrated using the velocity Verlet algorithm.

## 4. Conclusions

In conclusion, three types of magnetic nanoparticles coated with aminated chitosan containing from one to three amino groups removed from the polymer chain were obtained. The reaction of chitosan amination on the surface of the nanoparticles was both traditionally carried out in a solvent, and also via a new method by grinding in a mortar. The extracted materials were also dried by two methods: in a vacuum oven and by lyophilization. The surface of the obtained materials was characterized without noticing the effect of the amination method or the method of drying on the porosity of the material, the size of the nanoparticles, and the magnetization. However, it was found that the material coated with chitosan containing two distanced amino groups (Fe_3_O_4_ CS-Et(NH_2_)_2_) significantly deviates from the other two types of nanoparticles. It was characterized by a lower specific surface area, lower porosity, and higher hydrophilicity. As a result of the theoretical calculations carried out using a molecular dynamics simulations, an attempt was made to explain these differences between the materials. The obtained results showed that the aminated chitosan covering Fe_3_O_4_ -CSEt(NH_2_)_2_ was characterized by a different configuration of the polymer chain controlled by intramolecular hydrogen bonds involving the unsubstituted amine group close to the backbone.

The results of HSA immobilization on the surface of the obtained nanoparticles showed that the method of drying nanoparticles has a key influence on HSA binding efficiency. For the Fe_3_O_4_- CSEt(NH_2_) and Fe_3_O_4_-CS-Et(NH_2_)_3_ aminated materials dried by lyophilization, the protein immobilization efficiency was 200% higher than for nanoparticles dried in a vacuum oven. Also, the amination method had an effect on the efficiency of immobilization, for materials obtained without a solvent, the yield was increased by about 35%. Unfortunately, similar results were not observed for Fe_3_O_4_-CSEt(NH_2_)_2_ nanoparticles. Neither the synthesis method nor the drying by lyophilization increased the HSA immobilization yield, which was very low compared to the remaining nanoparticles.

The obtained results allow to state that limiting the presence of solvent during synthesis and its effective removal from the material by lyophilization allows to increase its ability to ligands binding.

## Figures and Tables

**Table 1:** Characterization of the prepared magnetic nanoparticles surface and size.

Nanoparticles Type	Size [nm]	Polydispersity Index (PDI)	Amount of NH_2_ Groups [mM/g]	Surface Area [m^2^/g]	Mesopore Volume [cm^3^/g]
Fe_3_O_4_-CSEt(NH_2_)	22	0.199	3.15	98	0.0357
Fe_3_O_4-_CSEt(NH_2_)_2_	25	0.186	5.93	34	0.0206
Fe_3_O_4-_CSEt(NH_2_)_3_	29	0.134	8.34	84	0.0383

**Table 2:** Contact angle measurement results for modified chitosan.

Sample	Average Contact Angle [θ, °]		Surface Free Energy [mJ/m^2^]		
	Measuring Liquid				
	Glycerin	Diiodomethane	γ_s_	γ_d_	γ_p_
CS	82	56	30.70	27.46	3.23
CS-Et(NH_2_)	85	59	28.87	26.21	2.66
CS-Et(NH_2_)_2_	67	46	38.21	30.11	8.10
CS-Et(NH_2_)_3_	87	61	27.67	25.35	2.32

**Table 3:** Results of HSA immobilization on magnetic nanoparticles Fe_3_O_4-_CSEt(NH_2_)_1–3._

Nanoparticles Type	HSA Loading [mg/g]		
	Amination Method	Dried in Vacuum	Dried by Lyophilization
Fe_3_O_4_-CSEt(NH_2_)	in solution	58.52	146.46
	solvent free	89.32	150.24
Fe_3_O_4-_CSEt(NH_2_)_2_	in solution	20.12	20.67
	solvent free	25.10	25.68
Fe_3_O_4-_CSEt(NH_2_)_3_	in solution	73.56	204.72
	solvent free	98.36	210.32
